# Skin Carotenoid Score as a Potential Early Biomarker of Metabolic Syndrome Risk in Adolescents

**DOI:** 10.3390/nu18020337

**Published:** 2026-01-21

**Authors:** Giuseppina Augimeri, Luca Gelsomino, Marco Germanò, Giovanni Tripepi, Daniela Bonofiglio, Renzo Bonofiglio

**Affiliations:** 1 Department of Pharmacy and Health and Nutritional Sciences, University of Calabria, Via P. Bucci, Arcavacata di Rende, 87036 Cosenza, Italy; luca.gelsomino@unical.it (L.G.); daniela.bonofiglio@unical.it (D.B.); 2 I.T.S. Nuove Tecnologie della vita A. Volta di Palermo—ITS Academy, 90123 Palermo, Italy; marcogermano23@gmail.com; 3 Consiglio Nazionale Delle Ricerche-Istituto di Fisiologia Clinica (CNR-IFC), Via Vallone Petrara snc, 89124 Reggio Calabria, Italy; gtripepi@ifc.cnr.it; 4 Centro Sanitario, University of Calabria, Arcavacata di Rende, 87036 Cosenza, Italy; 5 Department of Nephrology, Dialysis and Transplantation, “Kidney and Transplantation” Research Centre, Annunziata Hospital, 87100 Cosenza, Italy; rbonofi@gmail.com

**Keywords:** metabolic syndrome, Mediterranean Diet, skin carotenoid score, Veggie Meter^®^, adolescents

## Abstract

**Background/Objectives:** The increasing prevalence of overweight and obesity in adolescents represents a major global health concern. Adolescent weight gain frequently shows additional metabolic risk factors, including insulin resistance, hypertension, and dyslipidemia, whose co-occurrence defines the metabolic syndrome (MetS). Adherence to a healthy dietary pattern, such as the Mediterranean Diet (MD), has been shown to reduce the metabolic risk among adolescents. Skin carotenoid score has emerged as an objective and non-invasive indicator of MD adherence; however, its relationship with a cluster of metabolic parameters which characterize the MetS, including the triglyceride levels, diastolic blood pressure, and waist circumference, remains poorly explored. Here, we investigated the role of skin carotenoid score as an early biomarker of metabolic syndrome risk in adolescents. **Methods:** A sample of 634 healthy adolescents underwent anthropometric and clinical measurements, blood sample collection, and evaluation of the MD adherence by the Mediterranean Diet Quality Index for Children and Adolescents (KIDMED) questionnaire and the skin carotenoid levels by the Veggie Meter^®^. Student’s *t*-test, chi-square test, Pearson correlation, and the multivariable linear regression model were used for analyses. **Results:** Participants had a mean BMI Z-score of 0.02 ± 1.01; the metabolic serum profile and the cardiovascular parameters were within the normal range. Mean KIDMED and skin carotenoid scores were 5.21 ± 2.56 and 357 ± 96.58, respectively. Skin carotenoids were positively associated with height (*p* = 0.02), while they were inversely associated with weight (*p* = 0.008), BMI Z-score (*p* < 0.0001), diastolic blood pressure (*p* = 0.013), and triglycerides (*p* = 0.003). Moreover, the carotenoid score was positively associated with male gender and KIDMED score and negatively associated with waist circumference and triglyceride levels in multivariable regression analyses. **Conclusions:** Our results suggested the potential application of skin carotenoid score as a complementary biomarker for the early identification of adolescents at increased metabolic risk.

## 1. Introduction

Metabolic syndrome (MetS), a multifactor disease characterized by abdominal obesity, dyslipidemia, hypertension, and insulin resistance, represents a public health concern worldwide [1]. Different genetic and environmental factors have been shown to contribute to the onset of MetS. For instance, several polymorphisms have been found to increase the risk of MetS, including those on the Fat Mass and Obesity-Associated (*FTO*) [2], the Cholesteryl ester transfer protein (*CETP*) [2], and the Apolipoprotein A5 (*APOA5*) genes [3]. However, since genetic variation cannot be changed, interventions on the environmental risk factors, including diet and lifestyle, are usually taken into account to reduce the prevalence of MetS. Interestingly, several studies have demonstrated that the mother’s health and lifestyle during pregnancy influence the development of MetS in newborns. Indeed, obesity, hypertension, and hyperglycemia during pregnancy have been found as risk factors for MetS in children [4,5,6,7,8]. Moreover, intrauterine nutrition alters gene expression through epigenetic mechanisms [9], leading to the development of MetS. A sedentary lifestyle, diets enriched in fats, insufficient sleep, and inflammation have also been found as other modifiable risk factors of MetS [10,11]. Indeed, sedentary behaviors and an unbalanced diet lead to obesity, which represents the first hallmark of MetS [12]. As fatty acids accumulate in the adipose tissue, increased lipogenesis and gluconeogenesis occur in the liver. In addition, active adipose tissue stimulates the release of pro-inflammatory cytokines, which contribute to obesity-related metabolic disorders. In this context, serum triglyceride and LDL cholesterol levels increase, whereas levels of HDL cholesterol decrease, linking MetS with other pathological conditions, including cardiovascular disease.

To date, metabolic syndrome in children and adolescents lacks standardized and widely accepted diagnostic criteria., which makes it difficult to evaluate the burden of this disease and develop a therapeutic strategy in the young population [13]. However, there is a clear consensus that weight management represents the gold standard for MetS treatment in children and adolescents.

High adherence to the Mediterranean Diet (MD) pattern has been demonstrated to decrease the prevalence of MetS [10,14]. Recently, a meta-analysis revealed that children with a high MD adherence have a significant lower body mass index (BMI) and waist circumference compared to those with a lower adherence, confirming the protective effects of the MD pattern against obesity outcome [15]. MD is based on a high intake of fruits, vegetables, and whole grain, a moderate intake of poultry and fish, and low consumption of red meat and sweets [16]. To date, the evaluation of the adherence to the MD is based on a food questionnaire, such as the Mediterranean Diet Quality Index for Children and Adolescents (KIDMED) [17]. Although this questionnaire has been widely validated to assess the adherence to the MD, it is linked with several biases [18], such as interviewer bias. Moreover, mothers are not fully captured when answering the KIDMED questionnaire since the children’s habits may differ depending on the different contexts at school or home [19]. Skin carotenoid score measured by the Veggie Meter^®^ has emerged as an objective non-invasive tool to assess the MD adherence. Indeed, our previous studies have revealed a positive association between skin carotenoid score and KIDMED [20], suggesting its potential use as objective biomarker to assess the MD adherence. Carotenoids are biomolecules found in fruits and vegetables [21], which represent the main foods of the MD. After ingestion, carotenoids are stored in different tissues, including skin, where they give an estimate of fruit and vegetable consumption within two weeks [22,23]. Limited studies are currently available on the association among skin carotenoid score and MetS parameters [24,25,26], whereas, to the best of our knowledge, a lack of literature has been found in the association among skin carotenoids, MetS, and adherence to the MD pattern, especially in young healthy populations. Here, we explore the relationship between the skin carotenoid score assessed by the Veggie Meter^®^, the MD adherence, and the metabolic risk factors in a cohort of healthy adolescents.

## 2. Materials and Methods

### 2.1. Participants

Participants were recruited from Scientific High School “G. B. Scorza” of Cosenza in Southern Italy in the context of the scientific project “Pre.Di.Re” (Prevenzione delle malattie renali) of the ASIT (Associazione Sud Italia Trapiantati) from January 2023 to June 2023. A total of 634 students (325 girls and 309 boys) aged between 14 and 19 years old underwent examinations. The objective of this cross-sectional study was explained to the participants. Parental written informed consent was obtained prior to adolescent enrollment in the “Pre.Di.Re” trial. The inclusion criteria required participants to be healthy individuals, recruited in the Scientific High School “G. B. Scorza” of Cosenza. This study was approved by the Italian Ethics Committee of the University of Calabria, Italy (#0075402 17 October 2022).

### 2.2. Anthropometric Parameters, Biochemical Measurements, and Blood Pressure

A detailed description of the anthropometric measurements has been reported elsewhere [20]. Briefly, height, weight, and waist circumference were gathered by trained personnel using established and standardized procedures. Body mass index (BMI) Z-score was calculated as previously reported [27].

Fasting venous blood samples were collected to obtain serum for evaluation of glucose, total cholesterol, high-density lipoprotein (HDL) and low-density lipoprotein (LDL) cholesterol, and triglycerides. Arterial pressure was measured two times after a 10 min resting period and the second blood pressure measurement was considered for the data analysis.

### 2.3. Assessment of the Mediterranean Diet Adherence and Family History

Adherence to the MD was assessed when administering the validated KIDMED test to all participants [17]. The KIDMED is composed of 16 items, with 4 questions on negative dietary habits, scored with a value of −1 each, and 12 questions denoting positive dietary habits scored with a value of +1 each. A KIDMED score ranging from <0 to ≤12 was obtained, with higher scores representing a higher adherence to the MD. The family history of type 2 diabetes and hypertension were assessed as risk factors for developing MetS in adulthood.

### 2.4. Measurement of Skin Carotenoid Content

Veggie Meter^®^ (Longevity Link Corporation, Salt Lake City, UT, USA), a spectroscopy-based device (http://www.longevitylinkcorporation.com/products.html, accessed on 18 November 2025), was used to measure skin carotenoids, according to the manufacturer’s instructions. The detailed procedure was explained elsewhere [20].

### 2.5. Statistical Analysis

Data were reported as the mean and standard deviation (SD). Statistical differences were evaluated using parametric tests (Student’s *t*-test, one-way ANOVA, followed by Tukey’s post hoc test and Chi-square test) in GraphPad-Prism 7.00 software program. Multivariable linear regression and Pearson’s correlation analyses were conducted using SPSS v.25 and GraphPad Prism 7, respectively. Statistical significance was set at *p* < 0.05.

## 3. Results

### 3.1. General and Anthropometric Characteristics of the Study Cohort

Participant characteristics are shown in Table 1. Males and females were similar with respect to age, whereas as expected the height (*p* < 0.0001), weight (*p* < 0.0001), and waist circumference (*p* < 0.0001) resulted significantly higher in boys than girls. Although the BMI Z-score was similar between girls and boys, the waist circumference/height ratio was significantly higher in girls than boys (*p* = 0.003). The metabolic serum profile resulted within the normal range, although gender-related differences were found. In particular, glucose levels (*p* < 0.0001) and the total/HDL cholesterol ratio (*p* = 0.0005) were significantly higher in boys than girls, whereas an opposite trend was observed for the total (*p* < 0.0001), LDL (*p* < 0.0001), and HDL cholesterol (*p* < 0.0001). The cardiovascular parameters were within the normal range with boys showing higher systolic (*p* < 0.0001) and pulse pressure (*p* < 0.0001), whereas girls showed higher diastolic (*p* < 0.0001). Family histories of diabetes and hypertension were 57 and 65%, with no differences between sexes. Detailed age- and sex-stratified general, anthropometric, and metabolic characteristics of the study population are reported in Supplementary Table S1.

### 3.2. Mediterranean Diet Adherence and Skin Carotenoid Score in the Study Population

A medium MD adherence was revealed by the KIDMED questionnaire in the whole group, with girls having a significantly higher adherence than boys (*p* = 0.00465). The mean skin carotenoid content measured by the Veggie Meter^®^ was 357 ± 96.58 in the entire sample, with significantly higher values in boys than in girls (*p* = 0.0002) (Table 2).

**Figure 1:**
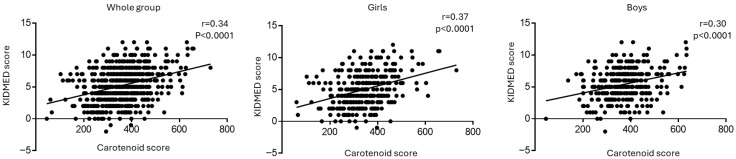
Correlations between carotenoid and KIDMED scores in the whole population, girls, and boys analyzed by Pearson’s correlation test.

Recently, we demonstrated that skin carotenoid score can be used as an objective method to assess the MD adherence in children and adolescents [20]. A positive association between carotenoid and KIDMED scores was found in the whole group and in both sexes (Figure 1).

### 3.3. Associations Between Carotenoid Score and Metabolic and Anthropometric Parameters

To analyze whether the carotenoid score is also associated with other variables, including general and anthropometric characteristics and the metabolic serum profile, we estimated the associations by Pearson’s correlation (Table 3).

**Figure 2:**
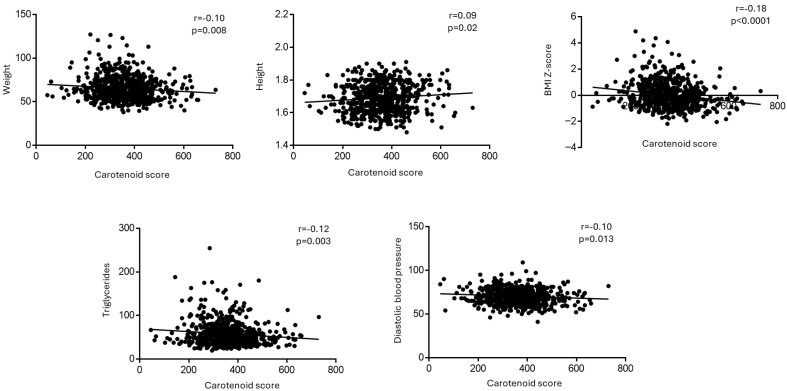
Correlations between carotenoid score and different parameters in the whole population analyzed by Pearson’s correlation test.

A positive association was found between the carotenoid score and height (*p* = 0.02), while negative ones were observed with weight (*p* = 0.008) and BMI Z-score (*p* < 0.0001). Moreover, the carotenoid score was negatively associated with diastolic blood pressure (*p* < 0.013) and triglycerides (*p* = 0.003) (Figure 2).

### 3.4. Association Between Skin Carotenoid Score and the Metabolic Syndrome Markers

Based on the univariable analysis, showing a correlation between carotenoid score and diastolic blood pressure as well as triglycerides, both components of the MetS, multivariable linear regression analysis was fitted with the carotenoid score as the dependent variable and the MetS parameters, including waist circumference, blood pressure, glucose, triglycerides, and HDL cholesterol, as independent variables. All models were adjusted for age and sex (Table 4). In Model 1, we found that the carotenoid score was significantly associated with males (*p* < 0.0001), waist circumference (*p* = 0.03), and triglyceride levels (*p*  =  0.02). In Model 2, we found that the family history of type 2 diabetes and hypertension did not influence the carotenoid score in our multivariable regression analysis.

Interestingly, including the KIDMED score among dependent variables of the MetS, we found that the carotenoid score was associated with MD adherence (*p* < 0.0001), along with males (*p* < 0.001), waist circumference (*p* = 0.02), and triglycerides (*p* = 0.04) (Model 3). The association also remains significant in the model adjusted for family disease history (Model 4).

## 4. Discussion

In 2021, 20% of adolescents aged 15–24 years were overweight or obese, worldwide [28]. Once obesity is established during childhood and adolescence, it rarely resolves in adulthood and it is associated with an increased risk of developing MetS and the related chronic degenerative diseases, including cardiovascular diseases, cancer, and infertility. To date, standard clinical parameters for MetS diagnosis in children and adolescents are not well defined. In addition, 50.7% of parents underestimate the weight of their overweight/obese children, complicating the early recognition and management of MetS. Identifying biomarkers that allow the detection of subclinical metabolic conditions in healthy young subjects represents a strategy for preventing the development of MetS. In this context, we enrolled healthy adolescents aged between 14 and 19 years old to investigate the relationship between the skin carotenoid score, MD adherence, and the cluster of metabolic abnormalities that characterize MetS, in order to validate the skin carotenoid score as a potential biomarker for the assessment of the MetS risk. We demonstrated that the skin carotenoid score, measured by the Veggie Meter^®^, is positively associated with waist circumference and triglycerides which represent two clinical parameters related to MetS.

Since skin carotenoids correlate with blood carotenoid levels, to evaluate their concentration in the human body [29], we assessed the skin carotenoid score by the Veggie Meter^®^ [30]. After the absorption, carotenoids are distributed in the tissues. Most carotenoids are stored in the adipose tissue, suggesting the negative correlation between skin carotenoid content and BMI, as we observed in our sample population. Particularly, we found a negative association between skin carotenoids and BMI Z-score, a standardized index used to categorize weight status in pediatric and adolescent populations. Our data also revealed that the carotenoid score is associated with the KIDMED score, suggesting that the lower carotenoid content depends on the adherence to the MD, based on the consumption of fruits and vegetables. We previously have demonstrated, according to other authors [31], that the skin carotenoid score measured by the Veggie Meter^®^ is positively associated with fruit and vegetable intake in different populations [32,33]. The data of this study further confirm that skin carotenoid levels reflect fruit and vegetable consumption and can be used as an objective tool to assess the adherence to the MD. Carotenoids have been widely studied for their beneficial properties, including the antioxidant and provitamin A activities and their ability to modulate gene expression and prevent the age-related macular degeneration. The relationship between skin carotenoids and the risk of several diseases, including the MetS, has been widely investigated. In a Japanese population, it has been found that skin carotenoids are lower in patients having MetS compared to the control. Moreover, a negative correlation was found between the lipid profile and the skin carotenoid levels [24]. An inverse correlation between the production of interleukin (IL)-β and IL6 by lipopolysaccharide peripheral blood mononuclear cells and plasma concentration of lutein–zeaxanthin has been observed in overweight women, suggesting that fruit and vegetable consumption affect the adiposity-related metabolic disturbances [25]. Furthermore, another study conducted on an adult Japanese population revealed a strong association between skin carotenoid scores and a lower prevalence of MetS [26]. In adolescents with MetS, blood carotenoid levels were lower than in healthy subjects, and inversely associated with the HOMA-IR and C-reactive protein (CRP) [34]. Moreover, it has been demonstrated that β-cryptoxanthin negatively correlated with cardiometabolic risk traits in children [35]. However, there is currently no evidence on the relationship between skin carotenoid levels and metabolic syndrome risk in healthy adolescent populations, which gave the rationale of our study. As mentioned above, skin carotenoid can be easily measured in an non-invasive way, which is particularly relevant for studies conducted in the young population. In multivariable regression analyses, we found that the skin carotenoid score is negatively associated with waist circumference and triglycerides, which represent two clinical parameters associated with the MetS. Indeed, the co-occurrence of high waist circumference and triglycerides, known as the hypertriglyceridemic waist phenotype, is strongly associated with increased cardiometabolic risk in non-overweight adolescents [36]. Although the family histories of type 2 diabetes and hypertension are associated with an increased likelihood of developing MetS in adulthood, we did not observe that these variables are associated with the carotenoid score. In contrast, as mentioned above, the carotenoid score was strongly associated with the KIDMED, along with triglyceride levels and waist circumference also in the multivariable regression analysis, emphasizing the role of the MD in promoting better health outcomes. The lack of awareness of MetS remains one of the greatest obstacles to its prevention and management in pediatric and adolescent populations. Moreover, the absence of standardized diagnostic criteria and treatment protocols for MetS in children and adolescents needs to be urgently well defined. Meanwhile, improving the dietary habits of the young population toward the MD and educating the adult population in a collaborative setting between families, schools, and society remains a public priority to reduce the burden of MetS.

The strength of our findings is that skin carotenoid score can have a clinical utility as a non-invasive and cost-effective tool for the early identification of MetS risk in healthy adolescents.

A limitation of this study is its cross-sectional setting, which does not allow the assessment of the causal association between skin carotenoid score and metabolic risk factors. Therefore, longitudinal observational and interventional studies are needed to determine whether the skin carotenoid score may predict the MetS risk in adolescents.

## 5. Conclusions

Our study has demonstrated the relationship between skin carotenoids, waist circumference, and triglyceride levels, highlighting the potential application of skin carotenoids as a complementary biomarker for MD adherence and early metabolic risk in adolescents, thereby supporting its use as a non-invasive approach in lifestyle-based prevention strategies.

## Figures and Tables

**Table 1:** Anthropometric measurements, metabolic profile, cardiovascular parameters and family history diseases of the study cohort.

	Whole Group (*n* = 634)	Girls (*n* = 325)	Boys (*n* = 309)	*p*-Value
Anthropometric Measurements				
Age (years)	16.63 ± 1.52	16.76 ± 1.46	16.49 ± 1.57	0.13
Weight (kg)	70.53 ± 14.01	60.37 ± 10.92	70.53 ± 14.01	<0.0001
Height (m)	1.69 ± 0.09	1.62 ± 0.06	1.75 ± 0.06	<0.0001
Waist circumference (cm)	83.09 ± 11.78	79.33 ± 10.50	87.03 ± 11.78	<0.0001
Waist circumference/Height ratio	0.49 ± 0.07	0.50 ± 0.006	0.49 ± 0.06	0.003
BMI Z-score	0.02 ± 1.01	−0.02 ± 0.96	0.04 ± 1.05	0.56
Metabolic serum profile (mg/dL)				
Glucose	87.54 ± 6.98	86.28 ± 7.16	88.87 ± 6.53	<0.0001
Total cholesterol	155.82 ± 27.28	162.95 ± 26.58	148.33 ± 25.99	<0.0001
HDL cholesterol	51.43 ± 10.78	54.62 ± 10.75	48.10 ± 9.76	<0.0001
LDL cholesterol	82.55 ± 21.56	85.05 ± 21.77	79.93 ± 21.05	0.003
Total/HDL cholesterol ratio	3.12 ± 0.70	3.03 ± 0.63	3.22 ± 0.76	0.0005
Triglycerides	57.89 ± 27.09	56.95 ± 25.59	58.88 ± 28.60	0.37
Cardiovascular Parameters (mmHg)				
Systolic pressure	115.17 ± 12.15	112.06 ± 11.04	118.44 ± 12.42	<0.0001
Diastolic pressure	70.44 ± 8.62	72.23 ± 8.02	68.56 ± 8.84	<0.0001
Pulse pressure	44.73 ± 12.04	39.84 ± 10.24	49.88 ± 11.65	<0.0001
Family history diseases				
Diabetes (n, %)	362, 57	195, 54	167, 46	0.13
Hypertension (n, %)	418, 65	221, 53	197, 47	0.26

BMI: body mass index, HDL: high-density lipoprotein, LDL: low-density lipoprotein, n: number of subjects.

**Table 2:** KIDMED and carotenoid scores in the total sample and by gender.

	Whole Group (*n* = 634)	Girls (*n* = 325)	Boys (*n* = 309)	*p*-Value
Carotenoid score	357 ± 96.58	343.2 ± 97.82	371.6 ± 93.22	0.0002
KIDMED score	5.21 ± 2.56	5.41 ± 2.46	5.02 ± 2.64	0.0465

KIDMED: Mediterranean Diet Quality Index for Children and Adolescents.

**Table 3:** Associations of carotenoid score with metabolic and anthropometric parameters.

	r	*p*-Value
Age	0.019	0.628
Weight	−0.10	0.008
Height	0.09	0.020
Waist circumference	−0.06	0.100
Waist circumference/Height ratio	−0.06	0.29
BMI Z-Score	−0.18	<0.0001
Glucose	0.06	0.154
Total cholesterol	0.005	0.891
HDL cholesterol	0.04	0.350
Total cholesterol/HDL ratio	−0.06	0.137
LDL cholesterol	−0.01	0.773
Triglycerides	−0.12	0.003
Systolic blood pressure	−0.01	0.721
Diastolic blood pressure	−0.10	0.013
Pulse pressure	0.06	0.157

BMI: body mass index, HDL: high-density lipoprotein, LDL: low-density lipoprotein.

**Table 4:** Multiple regression analyses of factors associated with the carotenoid score.

	Model 1		Model 2		Model 3		Model 4	
	β (CI 95%)	*p*-Value	β (CI 95%)	*p*-Value	β (CI 95%)	*p*-Value	β (CI 95%)	*p*-Value
Age	0.05 (−2.0; 7.84)	0.24	0.04 (−2.16; 7.76)	0.27	0.03 (−2.75; 6.54)	0.42	0.03 (−2.68; 6.70)	0.40
Males	0.19 (18.7; 54.46)	<0.0001	0.19 (19.17; 55.02)	<0.001	0.17 (16.58; 50.25)	<0.001	0.17 (16.96; 50.78)	<0.001
Waist circumference	−0.1 (−1.50; −0.08)	0.03	−0.1(−1.52; −0.1)	0.03	−0.1 (−1.48; −0.15)	0.02	−0.10 (−1.51; −0.17)	0.01
Systolic blood pressure	−0.01 (−0.83; 0.62)	0.78	−0.01 (−0.81; 0.64)	0.81	−0.03 (−0.93; −0.44)	0.49	−0.03 (−0.91; 0.46)	0.51
Diastolic blood pressure	−0.04 (−1.48; 0.51)	0.34	−0.04 (−1.51; 0.51)	0.32	−0.01 (−1.08; 0.80)	0.77	−0.02 (−1.13; 0.77)	0.71
HDL cholesterol	0.06 (−1.70; 1.30)	0.13	0.06 (−0.19; 1.28)	0.15	0.07 (−0.08; 1.30)	0.08	0.07 (−0.1; 1.29)	0.09
Triglycerides	−0.09 (−0.60; −0.04)	0.02	−0.09 (−0.60; −0.04)	0.03	−0.08 (−0.54; −0.01)	0.04	−0.08 (−0.54; −0.01)	0.04
Glucose	0.05 (−0.42; 1.75)	0.23	0.05 (−0.38; 1.81)	0.20	0.06 (−0.14; 1.91)	0.09	0.07 (−0.12; 1.94)	0.08
Family history of diabetes			0.02 (−11.35; 18.87)	0.62			0.01 (−11.44; 17.07)	0.70
Family history of hypertension			0.0018 (−15.82; 16.19)	0.98			0.02 (−11.18; 19.06)	0.61
KIDMED					0.33 (9.87; 15.43)	<0.001	0.33 (9.81; 15.41)	<0.001

HDL: high-density lipoprotein; KIDMED: Mediterranean Diet Quality Index for Children and Adolescents.

## Data Availability

The datasets used and/or analyzed in the current study are available from the corresponding author upon reasonable request.

## Supplementary Materials

The following supporting information can be downloaded at https://www.mdpi.com/article/10.3390/nu18020337/s1, Table S1: General and anthropometric characteristics of the study cohort stratified by age and sex.

## Abbreviations

APOA5	Apolipoprotein A5
ASIT	Associazione Sud Italia Trapiantati
BMI	Body Mass Index
CI	Confidence Interval
CETP	Cholesteryl Ester Transfer Protein
FTO	Fat Mass and Obesity-Associated Gene
HDL	High-Density Lipoprotein
IL6	Interleukin-6
KIDMED	Mediterranean Diet Quality Index for Children and Adolescents
LDL	Low-Density Lipoprotein
MD	Mediterranean Diet
MetS	Metabolic Syndrome
mmHg	Millimeters of Mercury
SD	Standard Deviation
