# Supplementary material for: Skin Carotenoid Score as a Potential Early Biomarker of Metabolic Syndrome Risk in Adolescents
Source: Nutrients. 2026 Jan 21;18(2):337. doi: 10.3390/nu18020337 (PMC12845373; doi:10.3390/nu18020337)
Supplement: Supplementary file 1 [file nutrients-18-00337-s001.zip › nutrients-4098936-supplementary.pdf]

Supplementary Table S1. General and anthropometric characteristics of the study cohort stratified by age and sex.

	**Whole group**<br>14-15y: 169<br>16-17y: 256<br>18-19y: 201	**Girls**<br>14-15y:74<br>16-17y: 144<br>18-19y:107	**Boys**<br>14-15y: 95<br>16-17y:121<br>18-19y:94	***p*-Value**
**Anthropometric Measurements**				
Weight (kg)	70.53±14.01	60.37±10.92	70.53±14.01	
14-15 years	62.65±13.61	57.5±9.80	66.61±14.81	<0.0001
16-17 years	65.71±13.29	61.82±11.7	70.35±13.63	<0.0001
18-19 years	67.06±13.39	60.4±10.26	74.65±12.51	<0.0001
p-value	§0.005	ns	§0.04	
Height (m)	1.69±0.09	1.62±0.06	1.75±0.06	
14-15 years	1.68±0.09	1.608±0.07	1.73±0.068	<0.0001
16-17 years	1.69±0.08	1.638±0.06	1.76±0.057	<0.0001
18-19 years	1.69±0.09	1.632±0.06	1.765±0.059	<0.0001
p-value	ns	ns	ns	
Waist (cm)	83.09±11.78	79.33±10.50	87.03±11.78	
14-15 years	80.21±11.71	75.78±10.11	83.65±11.76	<0.0001
16-17 years	85.01±12.15	82.13±11.56	88.44±11.97	<0.0001
18-19 years	82.98±10.84	78.02±8.07	88.62±10.87	<0.0001
p-value	ns	ns	ns	
Waist/Height ratio	0.49±0.07	0.50±0.006	0.49±0.06	
14-15 years	1.31±0.17	1.34±0.17	1.282±0.166	0.03
16-17 years	1.32±0.19	1.35±0.21	1.28±0.17	0.002
18-19 years	1.26±0.15	1.31±0.16	1.2±0.12	<0.0001
p-value	ns	ns	ns	
BMI Z-score	0.02±1.01	-0.02±0.96	0.04±1.05	
14-15 years	-0.15±1.03	-0.13±0.92	-0.16±1.12	ns
16-17 years	0.04±1.02	0.08±1.05	-0.01±0.10	ns
18-19 years	0.14±0.94	-0.02±0.86	0.33±0.10	0.009
p-value	ns	ns	ns	
**Metabolic serum profile** (mg/dl)				
Glucose	87.54±6.98	86.28±7.16	88.87±6.53	
14-15 years	87.04±6.75	86.03±7.10	87.82±6.39	ns
16-17 years	88.61±6.722	87.08±6.69	90.41±6.33	<0.0001
18-19 years	86.6±7.334	85.37±7.75	87.99±6.6	0.01
p-value	ns	ns	ns	
Total cholesterol	155.82±27.28	162.95±26.58	148.33±25.99	
14-15 years	152±24.7	158±22.09	147.3±25.7	0.005
16-17 years	154.6±27	163.6±25.87	143.8±24.29	<0.0001
18-19 years	160.6±29.05	165.5±29.96	155.1±27.1	0.01
p-value	§p<0.001, °0.0009	ns	°<0.0001	
HDL cholesterol	51.43±10.78	54.62±10.75	48.10±9.76	
14-15 years	51.28±10.58	54.5±10.37	48.78±10.09	0.0004
16-17 years	51.45±10.5	54.03±10.71	48.4±9.41	<0.0001
18-19 years	51.58±11.34	55.49±11.1	47.14±9.93	<0.0001

p-value	ns	ns	ns	
LDL cholesterol	82.55±21.56	85.05±21.77	79.93±21.05	
14-15 years	79.43±20.55	80.14±20.18	78.87±20.93	ns
16-17 years	81.61±20.61	86.07±20.94	76.33±18.97	<0.0001
18-19 years	86.38±23.07	87.08±23.55	85.57±22.6	ns
p-value	§0.0004	ns	°0.001	
Total/HDL cholesterol ratio	3.12±0.70	3.03±0.63	3.22±0.76	
14-15 years	3.06±0.73	2.89±0.55	3.184±0.82	0.01
16-17 years	3.10±0.64	3.079±0.65	3.107±0.63	ns
18-19 years	3.22±0.75	3.06±0.64	3.41±0.82	0.0008
p-value	ns	ns	ns	
Triglycerides	57.89±27.09	56.95±25.59	58.88±28.60	
14-15 years	58.11±28.92	57.58±27.93	58.52±29.81	ns
16-17 years	56.7±23.78	59.04±26.52	53.91±19.79	ns
18-19 years	59.17±29.54	53.68±22.34	65.41±35.13	0.005
	ns	ns	°<0.0001	
**Cardiovascular Parameters** (mmHg)				
Systolic pressure	115.17±12.15	112.06±11.04	118.44±12.42	
14-15 years	114.2±12.66	110.1±11.23	117.4±12.84	0.0001
16-17 years	114.7±12.14	112.1±11.05	117.9±12.67	<0,0001
18-19 years	116.7±11.69	113.4±10.79	120.4±11.62	<0,0001
p-value	ns	ns	ns	
Diastolic pressure	70.44±8.62	72.23±8.02	68.56±8.84	
14-15 years	69.28±7.84	70.64±7.98	68.22±7.61	0.04
16-17 years	71.4±8.94	73.54±7.94	68.84±9.41	<0,0001
18-19 years	70.1±8.714	71.56±7.93	68.47±9.29	0.01
p-value	ns	ns	ns	
Pulse pressure	44.73±12.04	39.84±10.24	49.88±11.65	
14-15 years	44.95±11.96	39.47±9.30	49.21±12.1	<0.0001
16-17 years	43.32±11.93	38.53±10.69	49.02±10.8	<0.0001
18-19 years	46.55±12.2	41.84±10.01	51.9±12.3	<0.0001
p-value	ns	ns	ns	
**Family history diseases**				
Diabetes (n, %)	362, 57	195, 54	167, 46	0.13
14-15 years	102, 28	46, 24	56, 34	0.67
16-17 years	150, 41	90, 46	60, 36	0.04
18-19 years	110, 30	59, 30	51, 30	0.20
p-value	ns	0.003	0.65	
Hypertension (n, %)	418, 65	221, 53	197, 47	0.26
14-15 years	116, 28	46, 21	70, 36	0.10
16-17 years	180, 43	103, 47	77, 39	0.17
18-19 years	122, 29	72, 33	50, 25	0.04
p-value	0.04	0.0005	0.17	

§ 14-15years vs 18-19 years; ° 16-17 years vs 18-19 years.
